# Immune regulatory networks and gut microbiota-associated targets in endometriosis: an integrative multi-omics and network analysis

**DOI:** 10.1515/biol-2025-1372

**Published:** 2026-09-08

**Authors:** Yan Liu, Shurong Liu, Chunyan Zhang, Lihua Pei, Jingyi Wu

**Affiliations:** Department of Gynaecology, Heping Hospital Affiliated to Changzhi Medical College, Changzhi, Shanxi Province, China; Department of Radiology, The Third People’s Hospital of Longgang, Clinical Institute of Shantou University Medical College (The Third People’s Hospital of Longgang District Shenzhen/Longgang Institute of Medical Imaging), Shantou University Medical College & The Third People’s Hospital of Longgang District, Shenzhen, Guangdong Province, China; Department of Chinese Medicine (Integrated Chinese and Western Medicine), Taizhou Municipal Hospital (Taizhou University Affiliated Municipal Hospital), Taizhou University, Taizhou City, Zhejiang Province, China

**Keywords:** endometriosis, gut microbiota, metabolites, MET, SELL, immune microenvironment`

## Abstract

Endometriosis is an estrogen-dependent disorder characterized by chronic inflammation, immune dysregulation, and altered hormone metabolism. Although gut microbiota-derived metabolites have been implicated in shaping immune microenvironments and lesion development, the underlying regulatory mechanisms remain incompletely understood. Here, we integrated multi-omics, protein-protein interaction (PPI), SHapley Additive exPlanations (SHAP), and single-cell transcriptomics to identify core targets and delineate their interactions with microbiota-derived metabolites, followed by molecular simulations to evaluate metabolite–target binding stability. In this study, we identified 61 differentially expressed genes (DEGs) associated with microbial metabolites. Among these, MET and SELL emerged as key hub genes, with MET showing a potentially protective association and SELL exhibiting a modest positive association with endometriosis risk. Specific gut microbes, including *Bacteroides*, *Parabacteroides*, and *Bifidobacterium*, along with metabolites such as palmitoylethanolamide and dihydroresveratrol, were implicated in modulating immune and inflammatory pathways relevant to lesion formation. Additionally, cell-cell communication analysis indicated that the 61 DEGs were enriched in the Macrophage migration inhibitory factor (MIF) and C-X-C motif chemokine ligand family (CXCL) signaling pathways, suggesting their potential importance in the endometriotic microenvironment. Computational simulations further indicated potential binding interactions between multiple metabolites and MET and SELL. These findings delineate a multi-layered regulatory framework linking gut microbiota, metabolites, molecular targets, and immune interactions, thereby providing a foundation for mechanistic studies and targeted therapeutic strategies.

## 1 Introduction

Endometriosis is an estrogen-dependent, chronic gynecological condition, characterized by the presence of tissue resembling the endometrium outside the uterus, often affecting the ovaries, fallopian tubes, and pelvic peritoneum [1]. Approximately 6–15 % of women in their reproductive years are affected by the disease, and its prevalence has been on the rise [2], 3]. Patients primarily experience symptoms such as painful periods, chronic pelvic pain, discomfort during sex, infertility, and gastrointestinal issues, which greatly diminish their quality of life and reproductive capabilities [4], 5]. The time from the appearance of symptoms to a confirmed diagnosis is often 7–10 years, primarily because of the varied symptoms and the absence of specific diagnostic markers [6]. Beyond its clinical implications, endometriosis is a major public health challenge, affecting mental health, the management of comorbidities, and the distribution of healthcare resources [1].

Studies have shown that the gut microbiota plays an important role in multiple key biological processes, including immune regulation, metabolism, inflammation, hormone regulation, and brain function [7], and is closely associated with the development and progression of inflammatory bowel disease [8], diabetes [9], polycystic ovary syndrome [10], ovarian cancer [11], and depression [12]. Endometriosis development is also strongly connected to the gut microbiota [13], 14]. Multi-omics studies have begun to characterize the relationship between gut microbiota and endometriosis-associated inflammation. Metagenomic and 16 S rRNA sequencing analyses have revealed significant alterations in microbial composition in patients [15], 16], while metabolomics studies have suggested that microbial-derived metabolites may be involved in modulating inflammatory and endocrine signaling pathways in endometriosis [17]. Clinical studies have shown that patients with endometriosis exhibit a decreased abundance of gut probiotics and an increased abundance of potential pro-inflammatory bacteria [5], which may influence the inflammatory environment and disease progression by modulating immune responses, inflammatory pathways, and estrogen metabolism.

Findings from animal model studies suggest that gut microbes and their metabolites can facilitate the growth of ectopic lesions and affect the distribution of immune cells [18]. Compared with healthy controls, the gut microbiota of endometriosis animal models is markedly altered, characterized by a decrease in Lactobacillus, an increase in Gram-negative bacteria, and a reduction in Bacteroides, accompanied by enhanced intestinal inflammation [19], 20]. Furthermore, increasing evidence indicates that microbiota-derived metabolites, including short-chain fatty acids, bile acids, and tryptophan metabolites, may regulate immune homeostasis and inflammatory signaling in endometriosis through host receptor-mediated pathways [21], 22]. In addition to modulating disease progression through microbial metabolites, the gut microbiota is also closely associated with host estrogen metabolism [23]. Based on this, the concept of the “estrobolome” has been proposed, referring to the capacity of gut microbes to regulate circulating estrogen levels via enzymes such as β-glucuronidase [24]. Microbial-derived β-glucuronidase enzymes encoded by the gut microbiota catalyze the deconjugation of estrogen glucuronides excreted via bile into the intestinal lumen, thereby converting inactive estrogen metabolites into their bioactive forms [25]. This process facilitates enterohepatic reabsorption of estrogens, leading to increased systemic estrogen availability, which may contribute to estrogen-dependent inflammatory conditions such as endometriosis [26]. Therefore, the gut microbiota serves as a key hub for immune, metabolic, and endocrine interactions in endometriosis, playing an important role in its pathogenesis and progression and providing new insights for understanding disease mechanisms and developing microbiota-targeted interventions.

Despite these advances, several important gaps remain. First, most studies have focused on either microbial compositional changes or metabolite associations, with limited integration of microbiota-derived metabolites and their downstream host target genes in endometriosis. Second, the functional links between metabolite-associated signals and disease-relevant host genes remain insufficiently characterized at a systems level. Therefore, an integrative framework is still needed to connect microbiota-derived metabolic signals with host gene regulation and to systematically identify their potential roles in endometriosis pathogenesis.

**Figure 1:**
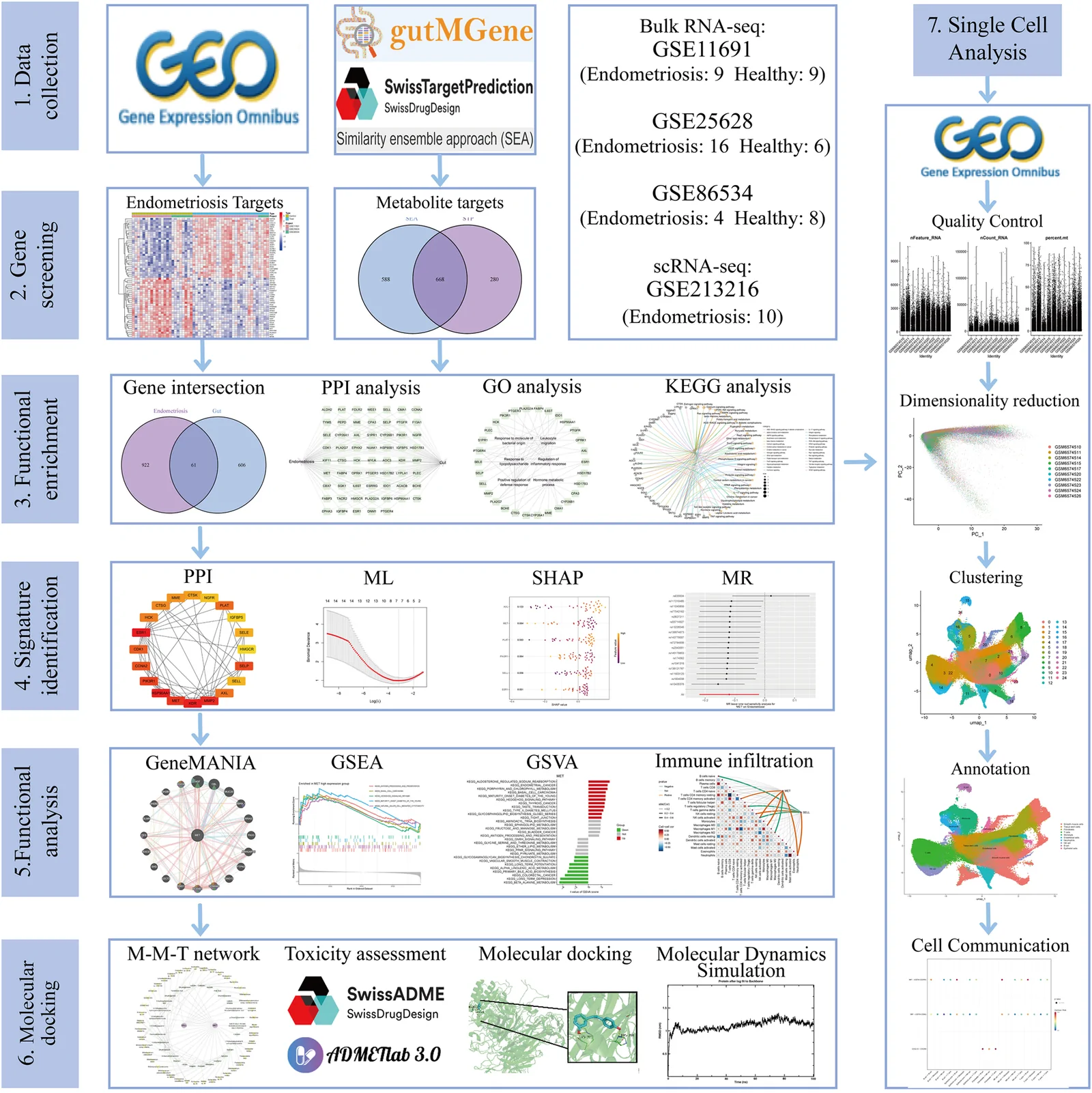
The workflow of this study.

To address these limitations, we developed an integrated multi-omics and network-based framework to systematically investigate how microbiota-derived metabolites regulate immune dysfunction and disease progression in endometriosis. By integrating metabolite-target interactions, host immune-related genes, and signaling pathways, we constructed a comprehensive interaction network and performed functional enrichment analyses to identify key regulatory processes. In addition, protein interaction networks and host–microbe interaction studies from previous reports support the existence of extensive transkingdom connectivity [27], 28], which we further explored through disease-specific network modeling. Single-cell RNA sequencing data were then used to resolve cell-type-specific immune communication patterns associated with key targets. Finally, molecular docking and molecular dynamics simulations were performed to assess the binding stability between prioritized metabolites and core protein targets. Therefore, this study provides a systems-level framework to bridge microbiota-derived metabolic signals with host immune regulation in endometriosis, offering new mechanistic insights and potential therapeutic targets. The overall workflow is shown in Figure 1.

## 2 Methods

### 2.1 Identification of potential targets for endometriosis

In this study, three gene expression datasets related to endometriosis were retrieved from the Gene Expression Omnibus (GEO) database to identify differentially expressed genes (DEGs). Specifically, GSE11691 (platform GPL96) includes 9 healthy tissue samples and 9 endometriosis samples; GSE25628 (platform GPL571) contains 6 healthy samples and 16 endometriosis samples; and GSE86534 (platform GPL20115) comprises 8 healthy samples and 4 endometriosis samples. All datasets were subjected to background correction, normalization, and batch effect adjustment prior to analysis. Differential expression analysis was performed using the limma package in *R*, with |log_2_ fold change (FC)| ≥ 0.585 and an adjusted *p* value < 0.05 set as the thresholds to identify DEGs associated with endometriosis.

**Ethical approval:** The conducted research is not related to either human or animal use.

### 2.2 Selection of gut microbial metabolites and targets

In this study, metabolite-target information related to gut microbiota was obtained from the gutMGene database (https://bio-computing.hrbmu.edu.cn/gutmgene/#/home), and the Simplified Molecular Input Line Entry System (SMILES) structures of metabolites were retrieved from the PubChem database (https://pubchem.ncbi.nlm.nih.gov/). For each gut microbiota–derived metabolite, potential human (“*Homo sapiens*”) targets were predicted using both the Similarity Ensemble Approach (SEA) (https://sea.bkslab.org/) and SwissTargetPrediction (STP) (http://swisstargetprediction.ch/). The predictions from these two methods were then compared using Venn analysis in R to identify overlapping, high-confidence targets for subsequent investigations.

### 2.3 Screening and PPI analysis of key targets

For the construction of the protein–protein interaction (PPI) network, core targets were first analyzed using the STRING database (https://cn.string-db.org/) and visualized in Cytoscape. The organism was set to human, and the minimum interaction score was set to medium confidence (0.4). Targets with the highest degree in the network were considered key nodes. Subsequently, the CytoHubba plugin was used to further screen core targets based on six algorithms: MCC, MNC, Degree, Closeness, Stress, and EPC, in order to identify the most influential proteins within the network.

### 2.4 Machine learning analysis

This study utilized four machine learning algorithms to pinpoint important predictive features: LASSO regression, neural network, support vector machine-recursive feature elimination (SVM-RFE), and random forest (RF). Feature selection and dimensionality reduction were primarily achieved using LASSO regression, with a random seed set at 123. To calculate feature importance and evaluate predictive performance, neural network and RF models were applied. The SVM-RFE analysis was executed using the e1071 package, with a random seed of 123. For the random forest model, the random seed was set to 123,456. Both R and Python environments employed standard cross-validation for model training and validation, with crucial features identified by their effect on model performance.

### 2.5 MR analysis of pQTLs

To assess potential causal relationships between protein expression and the study phenotype, two-sample MR was performed. SNPs associated with protein levels were selected at a significance threshold of *P* < 5 × 10^−6^. Independent SNPs were obtained by pruning for linkage disequilibrium, using *R* ^2^ < 0.001 and a minimum physical distance of 10,000 kb between variants. pQTL summary data were retrieved from the FinnGen database (https://www.finngen.fi/en/access_results) for the MR analysis.

### 2.6 Immune infiltration analysis

The immune cell composition of tissue samples was estimated using the CIBERSORT algorithm based on the LM22 leukocyte signature matrix. This approach was applied to quantify the relative proportions of 22 immune cell subtypes within each sample. The inferred immune cell fractions were subsequently used for comparative analysis between groups and correlation analysis with core genes.

### 2.7 Single-cell RNA sequencing analysis of endometriosis

Single-cell transcriptome data for 10 patients with endometriosis were retrieved from the GEO database, under accession number GSE213216, for this study. The raw data were quality-controlled to remove low-quality cells, as well as erythrocytes and ribosomal RNA transcripts, followed by log-normalization and data integration to eliminate batch effects. Uniform Manifold Approximation and Projection (UMAP) was used for dimensionality reduction, while cell clustering was achieved using the FindClusters function from the Seurat package. Cell type annotation was achieved using classical marker genes in combination with the SingleR package. Finally, the CellChat package was employed to construct the intercellular communication network within the endometriosis microenvironment, analyzing key signaling pathways and cell-cell interactions to elucidate the cellular landscape.

### 2.8 Drug-likeness and ADMET prediction of metabolites

In this study, SwissADME (http://www.swissadme.ch/) was used to evaluate the drug-likeness of metabolites associated with core targets. Since insufficient safety is a major reason for drug development failure, potentially leading to adverse effects such as hepatotoxicity (H-HT), carcinogenicity, drug-induced liver injury (DILI), and increased acute toxicity (LD50), ADMETlab 2.0 (https://admetmesh.scbdd.com/) was further employed to systematically predict and assess the absorption, distribution, metabolism, excretion, and toxicity (ADMET) properties of these metabolites.

### 2.9 Molecular docking

The three-dimensional structures of core target proteins were obtained from the AlphaFold Protein Structure Database (https://alphafold.ebi.ac.uk/). The predicted protein models were evaluated based on the predicted local distance difference test (pLDDT) scores provided by AlphaFold to assess structural confidence. Functional domains of the target proteins were annotated according to the UniProt database to assist the structural interpretation of docking results. Potential ligand-binding pockets were identified using CB-Dock2 based on its cavity detection algorithm. The docking search space was defined according to the predicted binding cavities, and docking poses with favorable binding energies and appropriate spatial configurations were selected for subsequent molecular dynamics simulations.

### 2.10 Molecular dynamics simulation of core target-metabolite complexes

Molecular dynamics (MD) simulations were performed on six gut-derived metabolites-core target complexes using GROMACS (version 2024) to evaluate the stability of the docked conformations and their dynamic interactions. The core target protein was parameterized with the AMBER ff14SB force field, and ligand topologies were generated using GAFF2. Each complex was solvated in a cubic box with TIP3P water, neutralized with ions, and supplemented with 0.15 M NaCl. After energy minimization and equilibration under NVT and NPT ensembles, production simulations were run for 100 ns with a 2-fs timestep. Trajectories were recorded every 10 ps, and key structural parameters, including RMSD, RMSF, Rg, and hydrogen-bond interactions, were analyzed using GROMACS tools.

## 3 Results

### 3.1 PCA data correction

**Figure 2:**
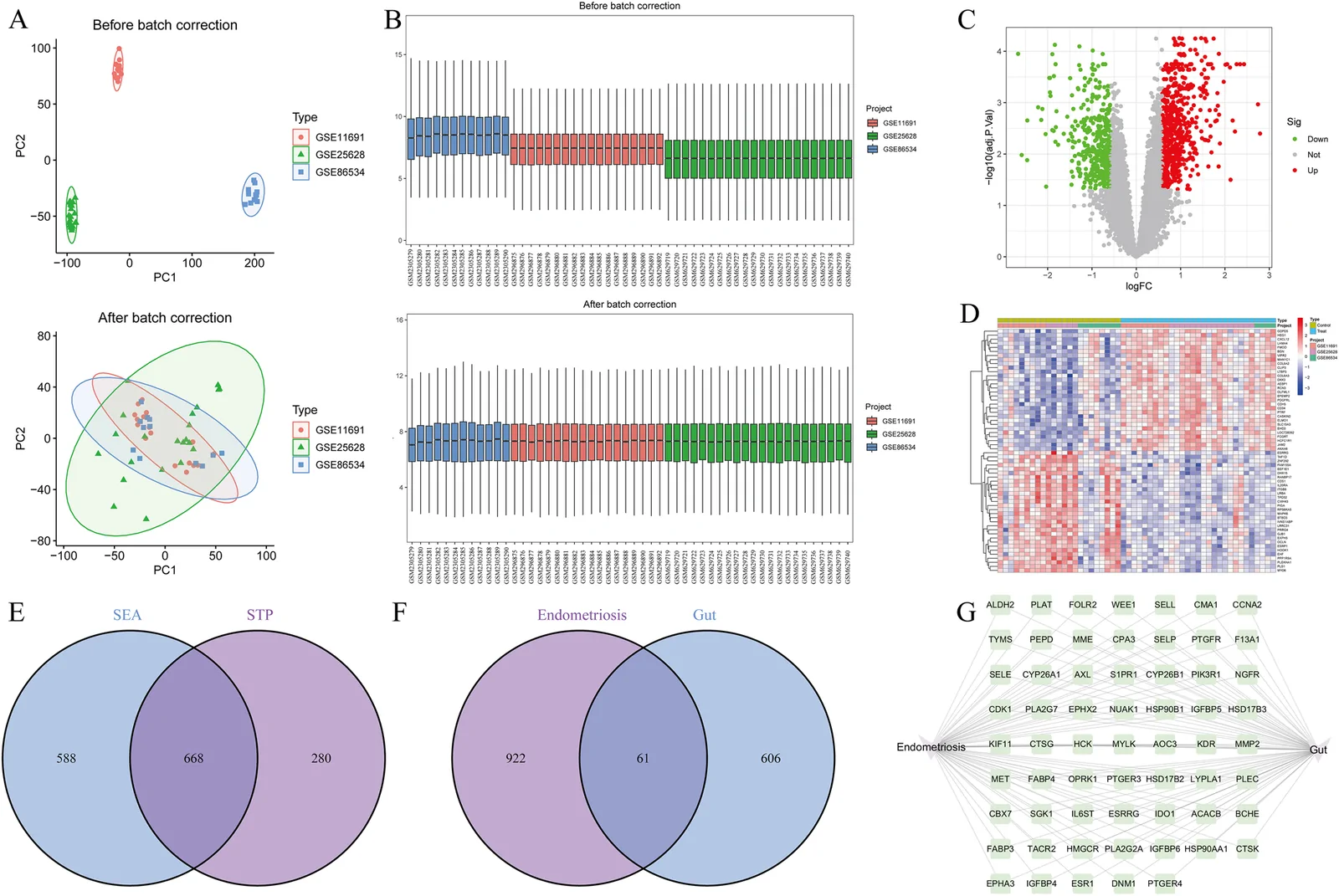
Differential expression analysis. (A, B) PCA analysis showing samples from different experiments before batch correction, with random shuffling, (C) heatmap of differentially expressed genes (DEGs) in endometriosis, (D) volcano plot of DEGs in endometriosis, (E) targets of gut microbiota metabolites, (F) common regulatory targets of the gut microbiota and endometriosis and (G) PPI network representing the common regulatory targets of gut microbiota and endometriosis.

Samples from different experiments were clearly divided before batch correction, highlighting a noticeable batch effect. The application of PCA resulted in a random distribution of samples from different experiments, thereby effectively reducing the batch effect (Figure 2A and B).

### 3.2 Identification of DEGs

Differential expression analysis identified a total of 983 DEGs (Figure 2C and D). Using the SEA and STP databases, 667 potential targets of gut microbiota-derived metabolites were predicted (Figure 2E). Among these, 61 targets were found to be associated with endometriosis (Figure 2F and G).

### 3.3 Functional enrichment analysis

**Figure 3:**
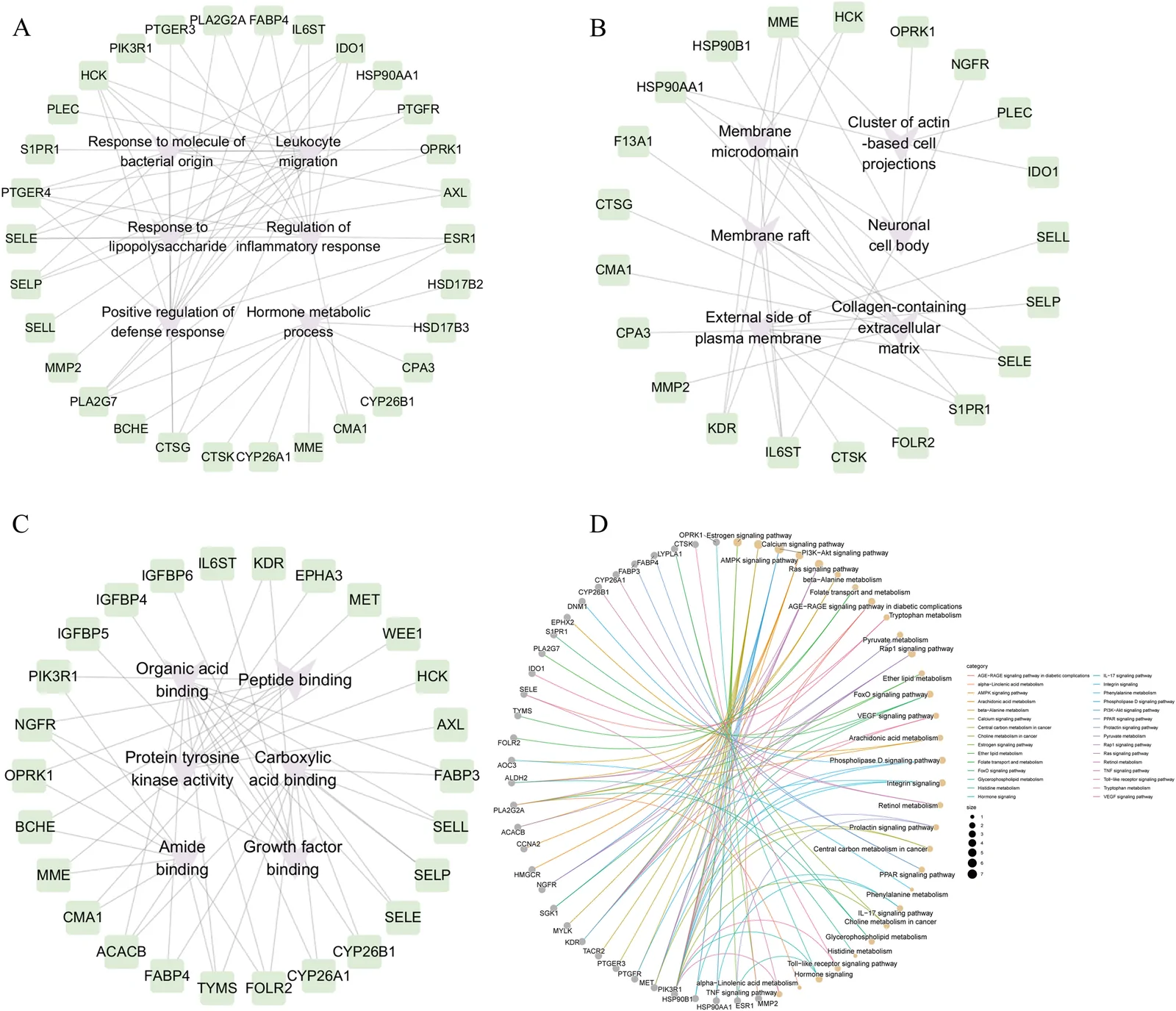
Functional enrichment analysis. (A) GO analysis of biological processes showing the top 6 significantly enriched terms along with their associated genes, (B) GO analysis of cellular components displaying the top 6 enriched cellular locations and corresponding genes, (C) GO analysis of molecular functions highlighting the top 6 enriched functional activities and related genes and (D) KEGG pathway enrichment analysis.

To investigate the biological functions of DEGs, GO analysis was performed, encompassing three categories: Biological Process (BP), Cellular Component (CC), and Molecular Function (MF). GO enrichment analysis facilitates a multi-level understanding of gene regulatory networks and their potential biological significance. The results showed that, at the BP level, DEGs were mainly enriched in hormone metabolism-related processes, leukocyte migration, regulation of inflammatory responses, positive regulation of defense responses, and responses to lipopolysaccharide and bacterial-derived molecules (Figure 3A), indicating a close association with immune and inflammatory responses as well as host defense mechanisms. At the CC level, DEGs were primarily localized to the external side of the plasma membrane, collagen-containing extracellular matrix, membrane rafts/microdomains, neuronal cell bodies, and actin-based cellular protrusions (Figure 3B), suggesting their potential involvement in cell surface signal transduction and cell-matrix interactions. At the MF level, DEGs were mainly enriched in organic acid and peptide binding, growth factor binding, and protein tyrosine kinase activity (Figure 3C), indicating that they may participate in immune regulation and cellular functional modulation through molecular binding and signal transduction.

KEGG pathway enrichment analysis revealed that DEGs were predominantly enriched in several classical signaling pathways, including the PI3K-Akt, MAPK, Ras, Rap1, and FoxO signaling pathways, as well as calcium signaling and phospholipase *D* signaling pathways, highlighting their important roles in cell proliferation, survival, migration, and signal transmission. In addition, these genes were significantly enriched in hormone-related signaling pathways (such as the estrogen signaling pathway and general hormone signaling pathways), the AMPK signaling pathway, and cell adhesion-related pathways mediated by integrins and IgSF CAMs, indicating their potential involvement in metabolic regulation, cell-cell and cell-matrix interactions, and immune-related processes. The enrichment of the AGE-RAGE signaling pathway further suggests that these genes may be closely associated with inflammatory responses and metabolism-related pathological processes (Figure 3D).

### 3.4 PPI network analysis

**Figure 4:**
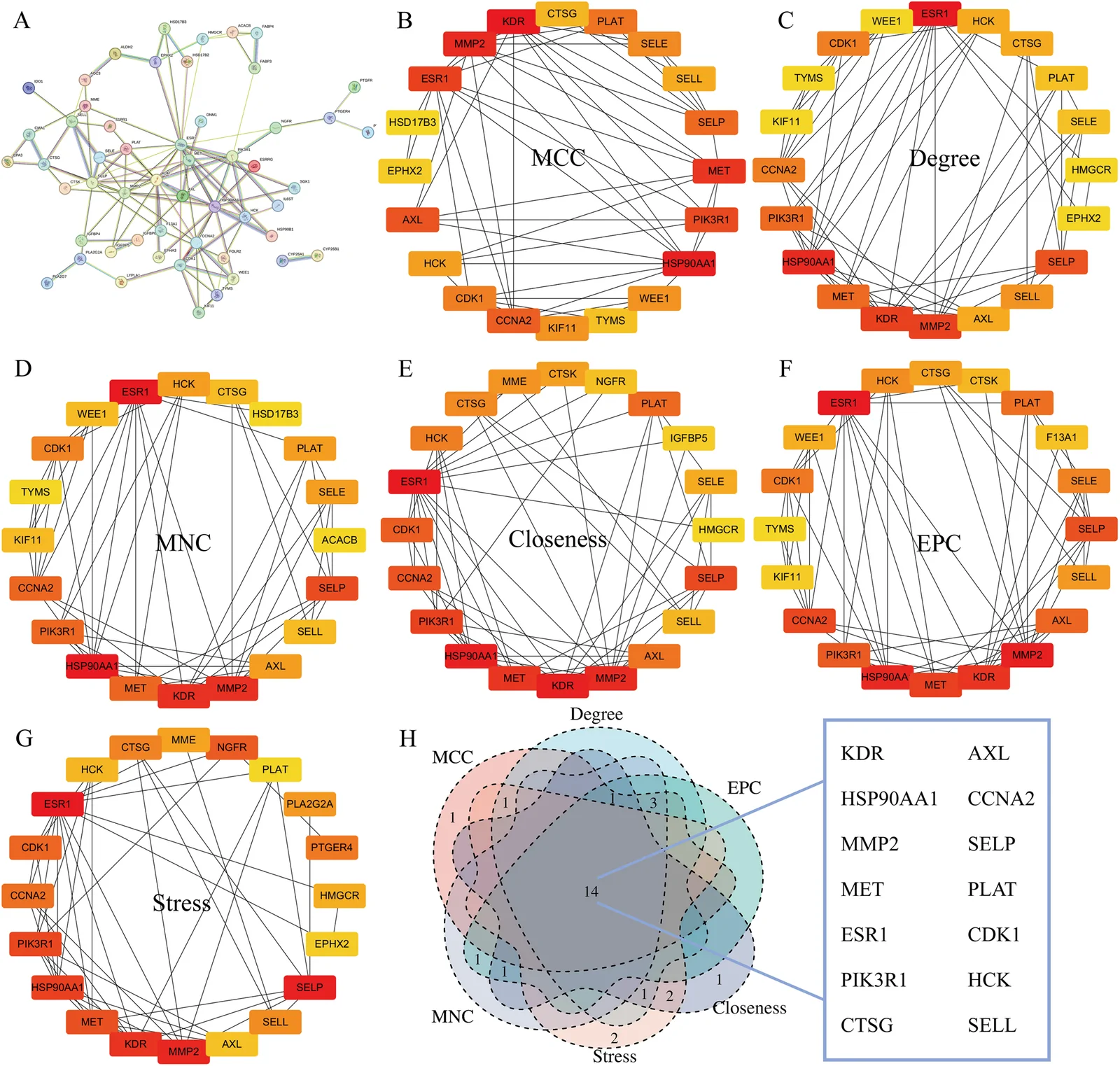
Key hub genes were identified through CytoHubba analysis. (A) Visualization of the protein–protein interaction (PPI) network for the DEGs using STRING; hub genes determined through multiple CytoHubba algorithms, including MCC, (B) degree, (C) DNC, (D) closeness, (E) EPC, (F) and stress, (G) to identify the most influential genes in the network and (H) venn diagram depicting the common hub genes identified across the different scoring methods, representing a consensus set of core genes for further functional exploration.

In this study, a PPI network composed of 61 DEGs was constructed and visualized based on the STRING database to intuitively illustrate the interactions among these genes (Figure 4A). To further identify potential key therapeutic targets, six network topological algorithms (MCC, Degree, MNC, Closeness, EPC, and Stress) were employed to score all nodes, assessing the centrality and importance of each gene within the network (Figure 4B–G). By intersecting the top 20 ranked genes identified by each algorithm, a total of 14 core target genes were ultimately identified. These genes exhibit high connectivity within the network and play critical regulatory roles (Figure 4H), providing priority candidate targets for subsequent studies.

### 3.5 Machine learning-based identification and validation of core genes

**Figure 5:**
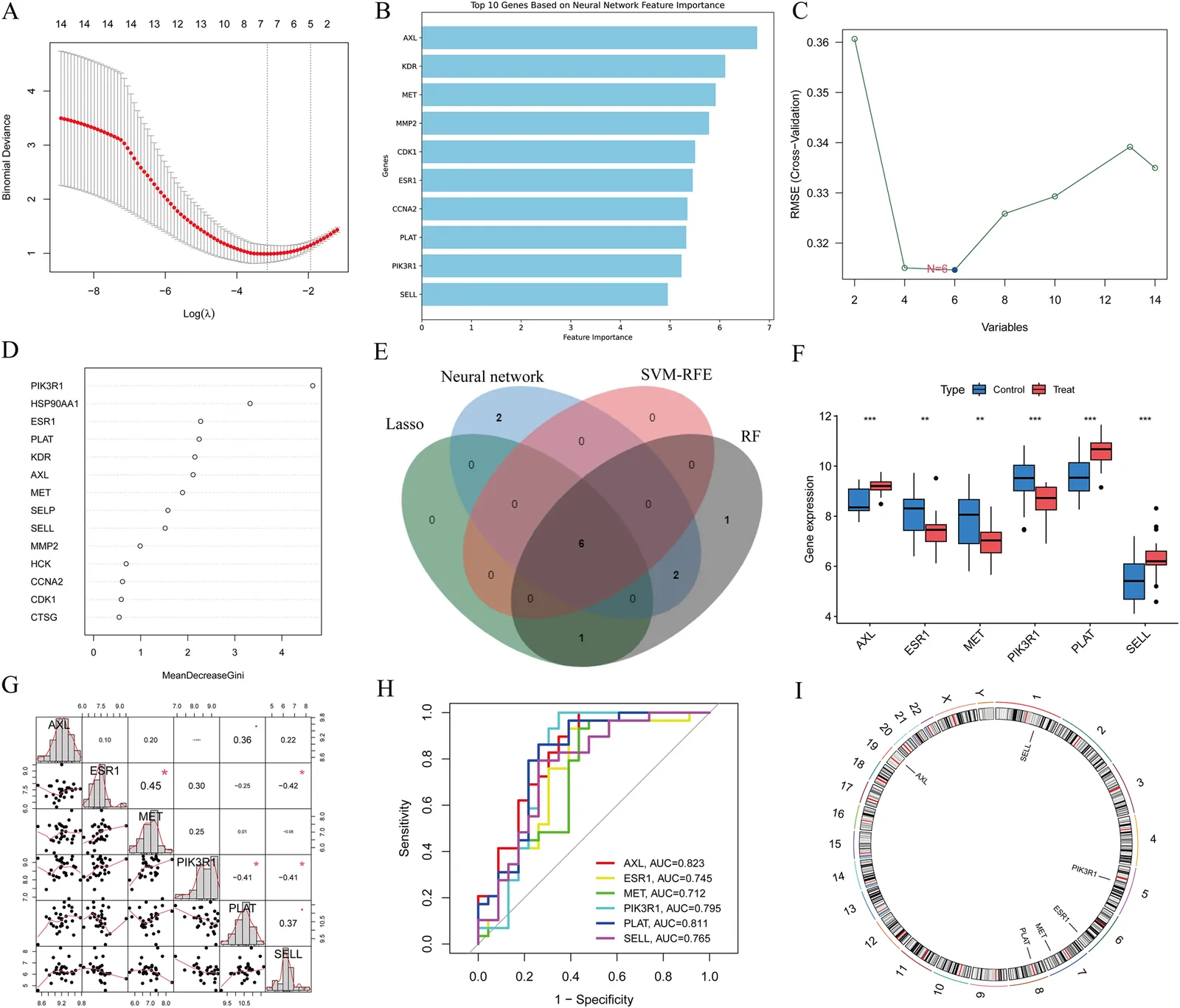
Identification and analysis of key genes using multiple approaches. (A) LASSO regression analysis for feature selection of candidate genes, (B) neural network modeling to evaluate gene importance and predictive contribution, (C) SVM-RFE predicted target genes, (D) random forest (RF) analysis for identifying genes with high predictive power, (E) venn diagram showing the intersection of candidate genes identified by LASSO, neural network, SVM-RFE, and RF, resulting in 6 key genes, (F) Boxplots illustrating the expression levels of DEGs, (G) expression relationships among the 6 key genes across samples, (H) diagnostic performance of individual genes assessed through ROC analysis and (I) chromosomal locations of the intersecting genes, mapped onto the corresponding chromosomes.

To further identify key genes, multiple machine learning approaches were applied to systematically screen the DEGs. LASSO regression analysis identified a total of seven core target genes (Figure 5A). A neural network model selected the top 10 genes based on feature importance scores (Figure 5B). The support vector machine-recursive feature elimination (SVM-RFE) method yielded 10 candidate genes (Figure 5C), while the random forest (RF) model likewise selected the top 10 genes ranked by importance (Figure 5D). By intersecting the results obtained from these four methods and visualizing the overlap using a Venn diagram, six common core target genes were ultimately identified (Figure 5E).

Boxplot analysis showed that all six core genes were significantly differentially expressed, with PLAT, SELL, and AXL being upregulated, while ESR1, MET, and PIK3R1 were downregulated (Figure 5F). Correlation analysis indicated a certain degree of co-expression among these genes (Figure 5G). ROC curve evaluation demonstrated that all genes exhibited high diagnostic performance (AUC > 0.7) (Figure 5H), suggesting their potential value in disease prediction. Finally, the chromosomal locations of these six core genes were analyzed (Figure 5I), providing a reference for subsequent functional studies.

### 3.6 Identification of key predictive features based on SHAP

**Figure 6:**
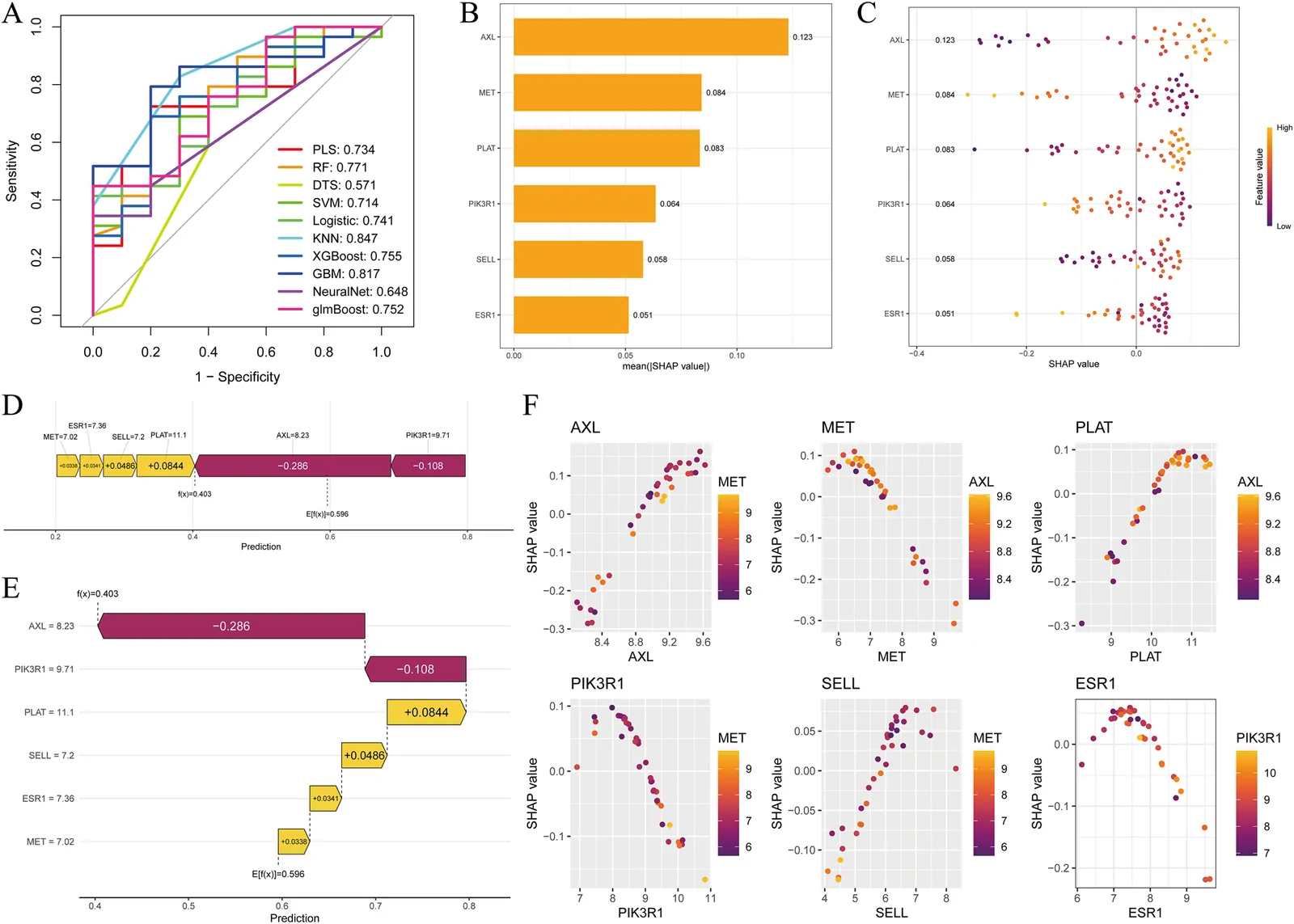
SHAP analysis. (A) ROC curves constructed using ten machine learning models to evaluate overall diagnostic performance, (B) bar chart showing the mean absolute SHAP values for each gene, with AXL contributing the most and ESR1 the least, (C) bee swarm plot illustrating the distribution of SHAP values for each gene, which can be used to interpret the positive or negative impact of genes on the prediction, (D) bar plot displaying the expression levels of the genes, (E) the waterfall plot shows the model prediction breakdown for a single sample, with the vertical axis representing gene expression levels and the horizontal axis representing prediction contributions. The larger the absolute value of the contribution, the greater the impact of the gene on the prediction and (F) the scatter plot illustrates the relationship between the expression level of a specific gene and its corresponding SHAP value. The horizontal axis represents gene expression, while the vertical axis shows the SHAP value. The color of the points ranges from purple to orange, reflecting the trend of expression level from low to high. This plot helps analyze the association between gene expression features and the model’s contribution.

SHAP was applied in this study to evaluate the impact of each gene feature on the endometriosis risk prediction by the top model, with the objective of uncovering the model’s decision-making strategy. The six essential target genes were analyzed using a set of 10 machine learning models and 10-fold cross-validation (Figure 6A). The results indicated that AXL was the most important predictive feature, whereas ESR1 contributed the least (Figure 6B). SHAP summary plot analysis showed that the impact of each feature on risk prediction varied in both direction and magnitude: some highly expressed risk genes significantly increased predicted risk, while others exhibited protective effects under certain conditions, partially offsetting overall risk (Figure 6C). Single-sample SHAP force plots demonstrated that the combined effect of multiple genes could substantially alter individual predictions (Figure 6D and E), suggesting that endometriosis risk is not determined by a single biomarker but by the interactions within a complex gene network. Moreover, these six core genes may have potential synergistic effects, indicating that they could collectively contribute to disease development and progression. The SHAP analysis highlights the critical roles of gene expression levels, microenvironmental factors, and potential gut microbial signals in the pathogenesis of endometriosis, providing a theoretical basis for future mechanistic studies and individualized risk prediction.

### 3.7 MR analysis identifies genetic causal targets

**Figure 7:**
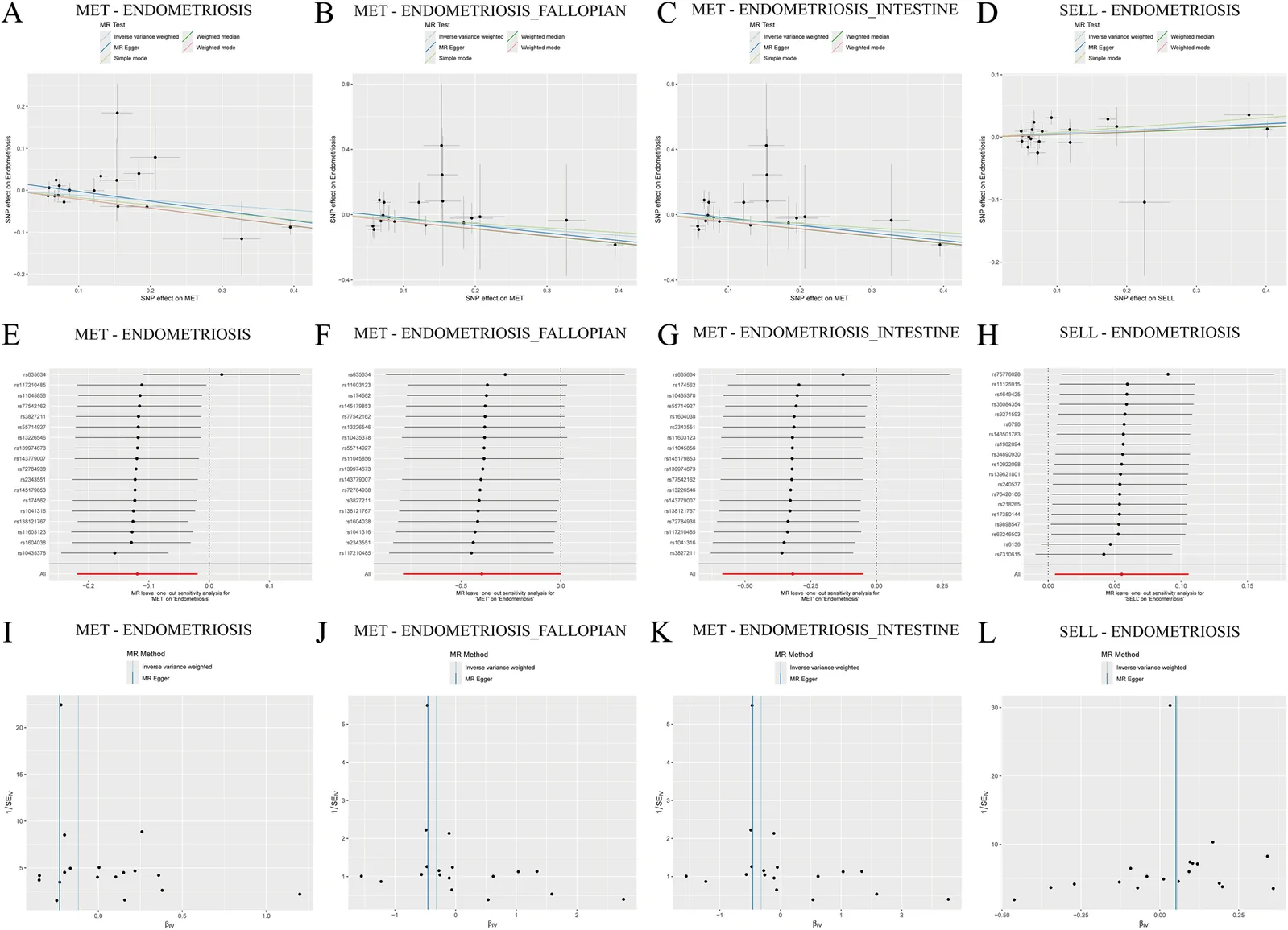
MR analysis based on protein quantitative trait loci (pQTL). (A) Funnel plot of MET on endometriosis, (B) funnel plot of MET on endometriosis-fallopian, (C) funnel plot of MET on endometriosis-intestine, (D) funnel plot of SELL on endometriosis, (E) leave-one-out plot of MET on endometriosis risk when leaving one SNP out, (F) leave-one-out plot of MET on endometriosis-fallopian risk when leaving one SNP out, (G) leave-one-out plot of MET on endometriosis-intestine risk when leaving one SNP out, (H) leave-one-out plot of SELL on endometriosis risk when leaving one SNP out, (I) scatter plot of the causal effect of MET on the risk of endometriosis, (J) scatter plot of the causal effect of MET on the risk of endometriosis-fallopian, (K) scatter plot of the causal effect of MET on the risk of endometriosis-intestine and (L) scatter plot of the causal effect of SELL on the risk of endometriosis.

MR analysis based on protein quantitative trait loci (pQTLs) was conducted to assess the genetic causal relationship between the core genes and endometriosis. Using the inverse-variance weighted (IVW) method as the primary criterion, among the six core genes, only MET and SELL showed significant genetic associations with endometriosis. After instrument selection and quality control, 18 independent SNPs were retained as instrumental variables for MET and 19 independent SNPs for SELL. Specifically, MET was significantly associated with overall endometriosis (*p* = 0.0194, OR = 0.888, 95 % CI: 0.803–0.981) (Figure 7A, E and I), and also exhibited significant or borderline associations with fallopian tube-type (*p* = 0.0495, OR = 0.673, 95 % CI: 0.453–0.999) (Figure 7B, F and J) and intestinal-type (*p* = 0.0197, OR = 0.727, 95 % CI: 0.557–0.950) (Figure 7C, G and K) endometriosis. SELL showed a statistically significant but modest association with overall endometriosis (*p* = 0.0309, OR = 1.057, 95 % CI: 1.005–1.111) (Figure 7D, H and L), indicating a weak positive genetic effect on disease risk. Although the effect size for SELL is modest, these findings suggest potential roles for MET and SELL in endometriosis susceptibility and provide preliminary evidence for further mechanistic and functional studies.

The MR robustness analysis showed that the Cochran’s *Q* test under the IVW method did not indicate significant heterogeneity, suggesting good consistency among the selected SNPs. The MR-PRESSO global test also did not detect any outlier SNPs, indicating that the results are unlikely to be driven by abnormal instrumental variables. In addition, the MR-Egger intercept test did not reveal significant directional horizontal pleiotropy. Meanwhile, the overall strength of the instrumental variables for MET and SELL was relatively high, with no evidence of weak instrument bias (Table 1). Taken together, the MR results for MET and SELL demonstrate good robustness and reliability.

**Table 1:** Comprehensive sensitivity and instrument strength analyses for MR results.

Outcome	Exposure	Heterogeneity test			Horizontal pleiotropy			Global test		*F*		
		Method	*Q*	*p*-Value	Egger intercept	se	*p*-Value	RSSobs	*p*-Value	Mean-F	Min-F	Max-F
Endometriosis	MET	IVW	38.709	0.096	0.020	0.011	0.076	66.203	0.350	115.579	31.336	1,034.491
Endometriosis_fallopian	MET	IVW	9.386	0.927	0.022	0.045	0.629	10.324	0.954	115.579	31.336	1,034.491
Endometriosis_intestine	MET	IVW	12.944	0.740	0.025	0.031	0.432	16.271	0.689	115.579	31.336	1,034.491
Endometriosis	SELL	IVW	16.879	0.531	0.001	0.005	0.885	20.450	0.530	236.022	29.735	3,575.392

### 3.8 Functional analysis of MET and SELL

**Figure 8:**
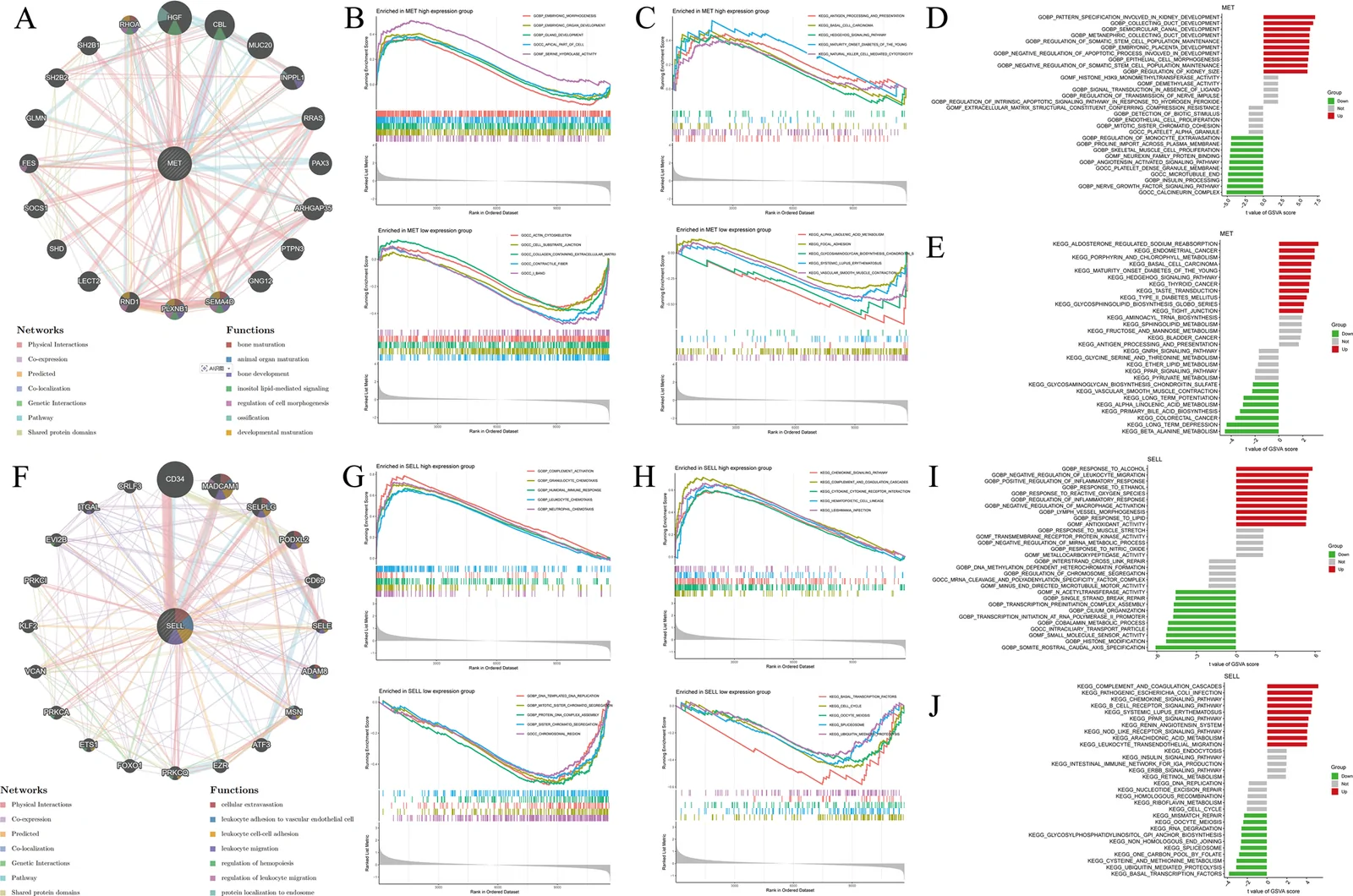
Functional analysis of MET and SELL. (A–E) GeneMANIA, GSEA, and GSVA analyses were performed to characterize the gene-gene interaction networks, enriched biological functions, and pathway activity patterns associated with MET, including GO terms, KEGG pathways, and sample-level functional scores and (F–J) the same analyses were conducted for SELL to evaluate its interaction network, functional enrichment profiles, and pathway activity patterns.

GeneMANIA analysis showed that MET is associated with skeletal and organ development, ossification, and regulation of cell morphology (Figure 8A). GSEA indicated that high MET expression is mainly enriched in immune, tumor, and developmental pathways, whereas low expression is linked to metabolism, extracellular matrix, and cytoskeleton–related pathways (Figure 8B and C). GSVA further suggested that high expression contributes to embryonic and epithelial development, while low expression affects immune regulation and metabolic homeostasis (Figure 8D and E). Therefore, MET plays a key role in tissue development, metabolism, and immune responses, with its expression level determining the activity of different biological processes.

GeneMANIA analysis showed that SELL is associated with leukocyte adhesion, migration, extravasation, and hematopoietic regulation (Figure 8F). GSEA indicated that high SELL expression is mainly enriched in immune, inflammatory, and complement-related pathways, whereas low expression is linked to cell cycle, basic metabolism, and protein degradation pathways (Figure 8G and H). GSVA further suggested that high expression promotes immune responses and leukocyte activation, while low expression affects transcription and cellular structural homeostasis (Figure 8I and J). Therefore, SELL plays a key role in immune regulation and inflammatory responses, with its expression level determining the balance between immune activity and metabolic state.

### 3.9 Immune infiltration analysis

**Figure 9:**
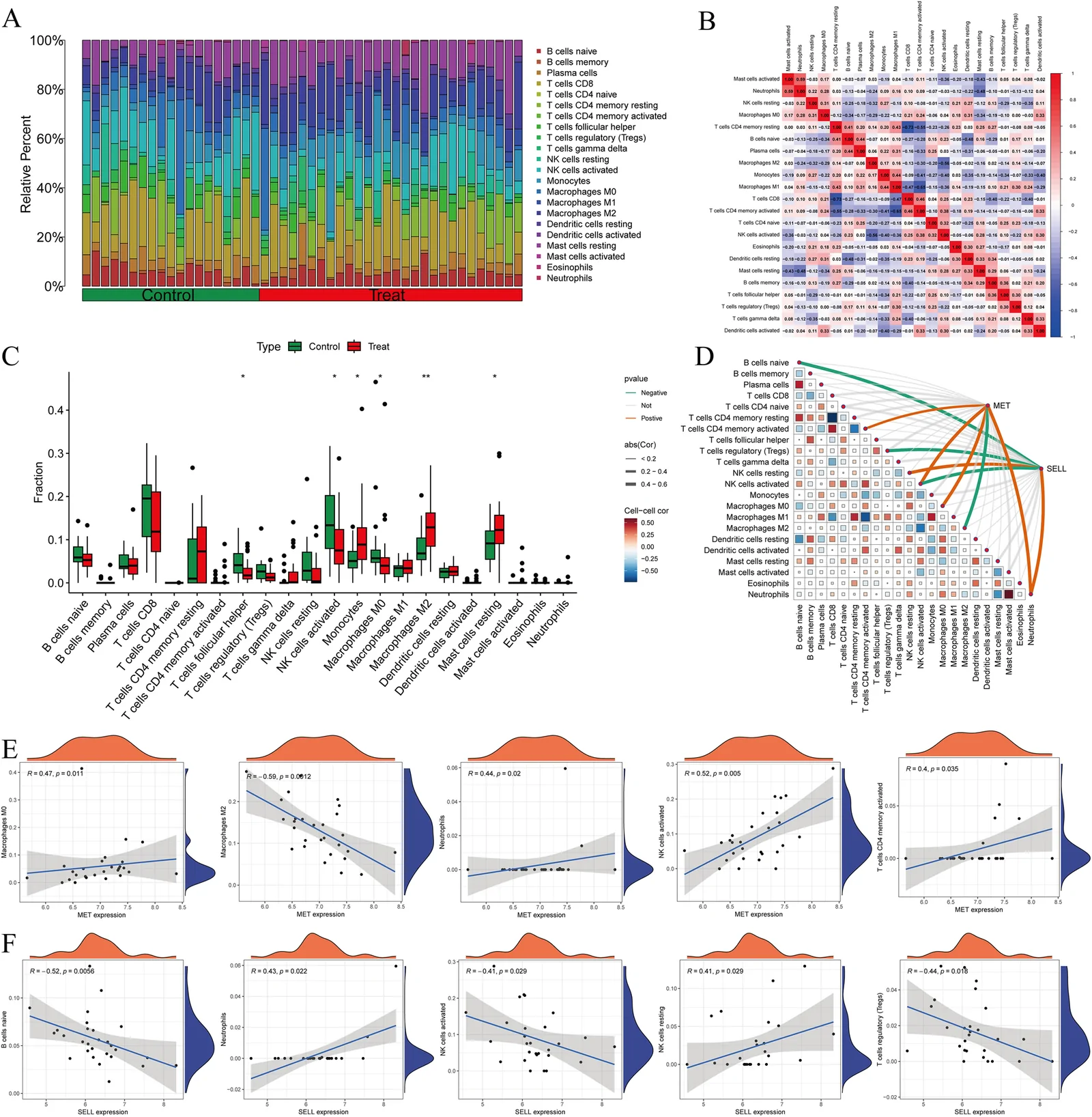
Immune infiltration analysis. (A) Bar plot showing the composition of immune cell types across samples, (B) correlation heatmap illustrating the relationships among different immune cell populations, (C) comparison of immune cell proportions between the control and endometriosis groups, (D) correlation between the expression of MET and SELL and various immune cell types and (E–F) correlation analysis of MET and SELL expression with five specific immune cell types associated with its expression.

To further explore the relationship between MET and SELL and the immune microenvironment, this study systematically evaluated different types of immune cells. Bar plots illustrated the distribution of immune cells in the experimental and control groups, while heatmaps revealed the interactions among immune cells (Figure 9A and B). Differential boxplot analysis showed that certain immune cells differed significantly between the two groups: M2 macrophages and monocytes were markedly increased in the experimental group, whereas M0 macrophages and helper T cells were higher in healthy controls (Figure 9C). The expression of MET was positively correlated with M0 macrophages and activated NK cells, but negatively correlated with M2 macrophages, as indicated by correlation analysis. Neutrophils were positively correlated with SELL expression, whereas regulatory *T* cells and naïve *B* cells were negatively correlated (Figure 9D–F). The findings suggest that MET and SELL might modify the immune microenvironment in endometriosis by changing the proportion or activity of certain immune cells, potentially having protective effects.

### 3.10 Single-cell analysis

**Figure 10:**
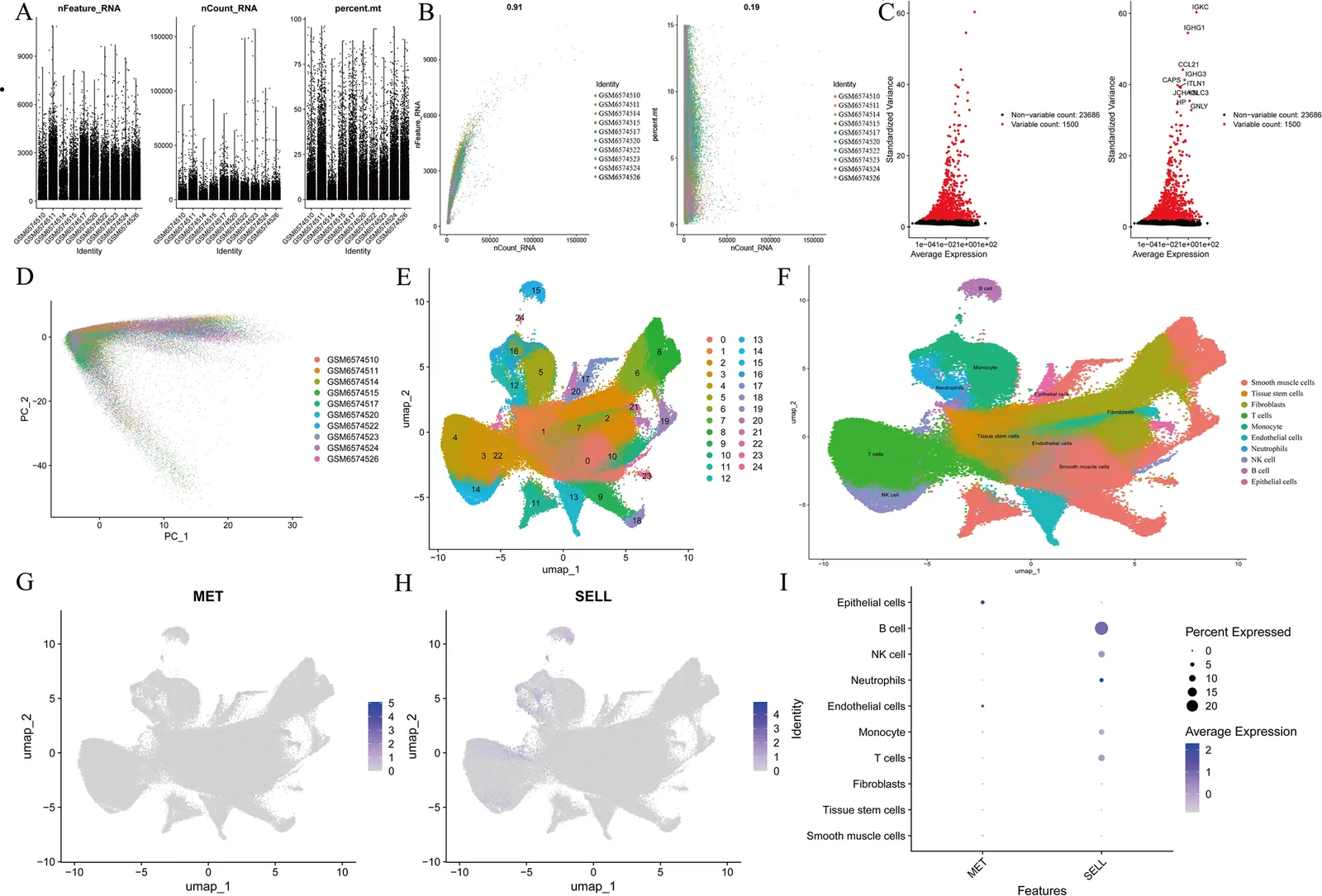
Single-cell quality control, dimensionality reduction, clustering, and annotation. (A–B) Quality control process showing the relationship between sequencing depth and detected gene counts (*R* = 0.91), and the proportion of mitochondrial gene expression (*R* = 0.19), indicating normal sequencing depth and no severe mitochondrial contamination, (C) selection of highly variable genes for downstream analysis, (D) principal component analysis (PCA) for dimensionality reduction, (E) identification of 25 clusters based on clustering analysis, (F) annotation using SingleR, resulting in 10 distinct cell subpopulations and (G–I) expression patterns of MET and SELL across the identified cell subpopulations.

In this study, single-cell RNA sequencing data from 10 patients with endometriosis were obtained from the GEO database, and low-quality cells were removed during quality control. Feature plots showed that the normalized gene expression values of the retained cells ranged from 0.19 to 0.91, indicating that certain genes were relatively highly expressed in specific cell types (Figure 10A and B). A total of 1,500 highly variable genes were identified (Figure 10C). Principal component analysis demonstrated good separation among the 10 individual samples, suggesting minimal batch effects (Figure 10D). UMAP dimensionality reduction subsequently identified 25 cell clusters, which were annotated into 10 cell subtypes using the SingleR package (Figure 10E and F). Further analysis revealed that MET was primarily expressed in epithelial cells, while SELL was expressed in *B* cells, NK cells, neutrophils, monocytes, and T cells (Figure 10G–I), suggesting that these genes may play key regulatory roles in the immune microenvironment.

### 3.11 Intercellular communication analysis in the immune microenvironment of endometriosis

**Figure 11:**
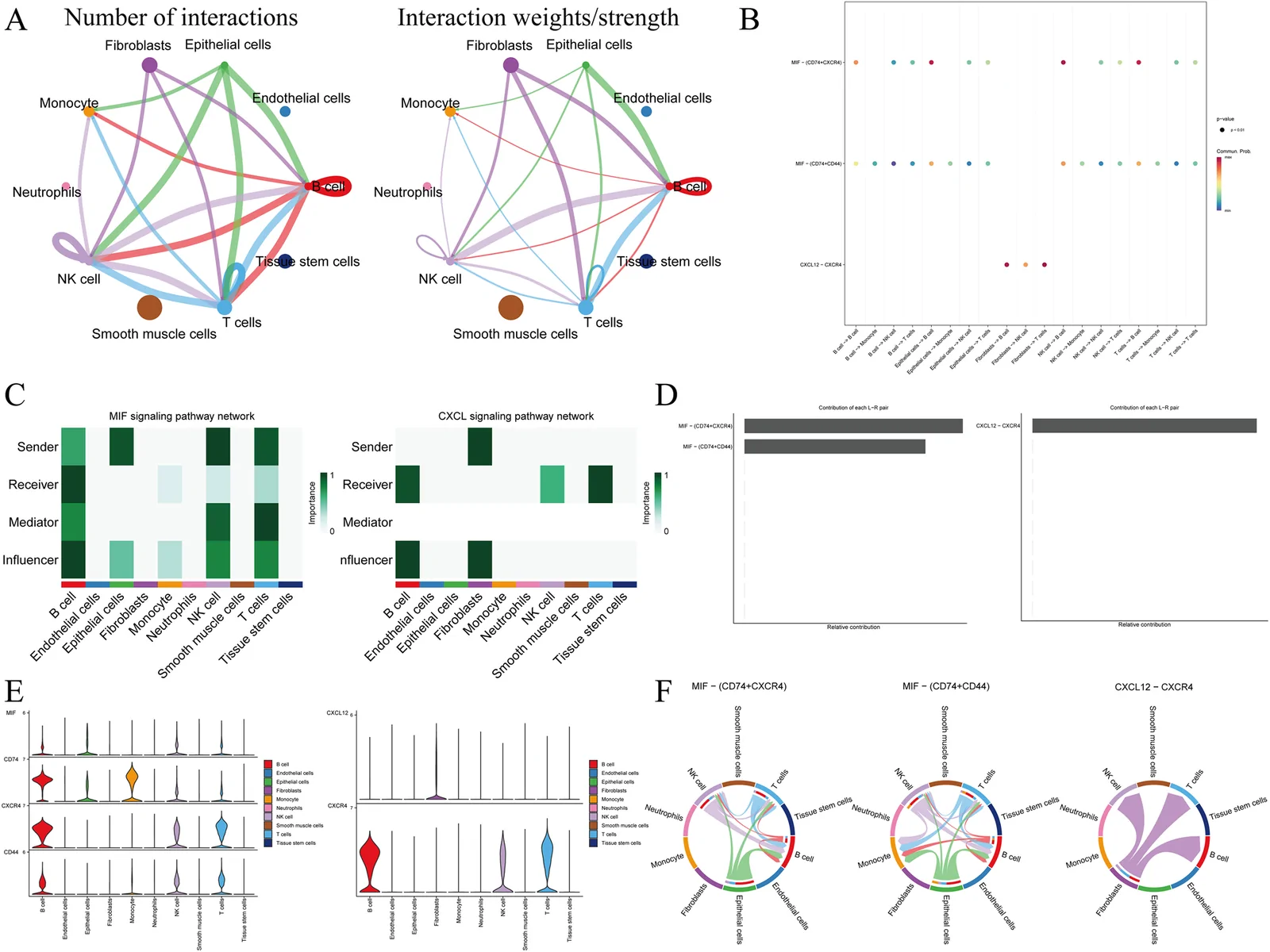
Cell-cell communication analysis. (A) Number and strength of interactions among cell types, representing the overall communication network, (B) bubble plot visualizing cell–cell communication between different cell populations, (C) network representation of the MIF and CXCL signaling pathway, showing interactions among participating cells, (D) contribution of ligand-receptor pairs to the MIF and CXCL signaling pathway, (E) expression patterns of genes involved in the MIF and CXCL signaling pathway across cell types and (F) summary of key ligand-receptor interactions within the MIF and CXCL signaling pathway.

This study constructed a cell-cell communication network within the endometriosis immune microenvironment to reveal its regulatory characteristics. The analysis showed that the MIF and CXCL signaling pathways are the primary communication axes (Figure 11A and B). In the MIF pathway, epithelial cells and NK cells act as the main signal senders, subsequently influencing other immune subpopulations such as B cells. In the CXCL pathway, fibroblasts serve as the primary signal senders, affecting immune subpopulations including B cells and T cells (Figure 11C). These two pathways are mainly mediated by three key ligand-receptor pairs: MIF-(CD74 + CXCR4), MIF-(CD74 + CD44), and CXCL12-CXCR4 (Figure 11D). Further analysis indicated that CXCL signaling primarily mediates communication between fibroblasts and immune cells, whereas MIF signaling mainly mediates interactions between epithelial cells and immune cells (Figure 11E and F). This suggests that these receptors play critical roles in regulating immune cell responses to microenvironmental signals and shaping the overall immune microenvironment.

### 3.12 Construction and analysis of a microbiota-metabolite-target regulatory network

**Figure 12:**
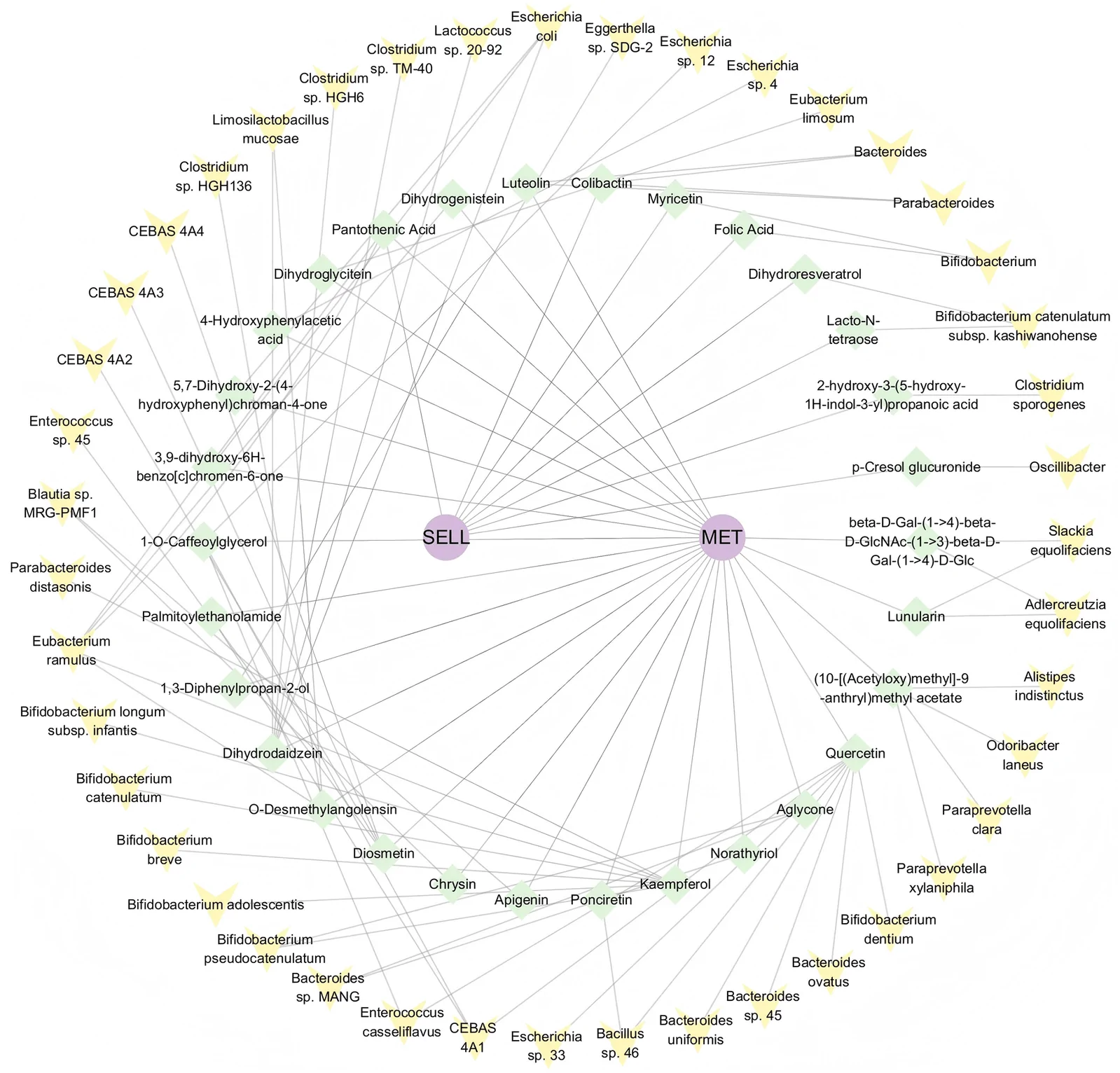
A gut microbiota-metabolite network centered on MET and SELL. In the network, purple nodes represent core target genes, green nodes represent microbial metabolites, and yellow nodes represent gut microbial species.

In this study, a microbiota-metabolite-target regulatory network was constructed and analyzed using Cytoscape. The network comprised 76 nodes, including 43 gut microbial species, 31 key metabolites, and the core targets SELL and MET (Figure 12). These findings further highlight the pivotal intermediary roles of MET and SELL in mediating microbiota-host interactions.

### 3.13 In silico ADME and toxicity evaluation for screening potential candidate metabolites

Since physicochemical properties alone cannot fully reflect the safety of metabolites, this study further performed toxicological predictions for 31 metabolites using the ADMElab platform. The results identified six metabolites as relatively safe with promising developmental potential, warranting further investigation (Table 2). Subsequently, in silico ADME evaluations of these six metabolites showed that all possessed acceptable physicochemical properties and potential bioactivity (Table 3).

**Table 2:** The evaluation of toxicity on key metabolites.

Compound	hERG blockers	H-HT	DILI	Carcinogencity	LD50 (mg/L)
Dihydroresveratrol	Non-blocker	Negative	Negative	Negative	5.017
Lunularin	Non-blocker	Negative	Negative	Negative	5.198
Dihydroglycitein	Non-blocker	Negative	Negative	Negative	6.764
Palmitoylethanolamide	Non-blocker	Negative	Negative	Negative	4.236
Pantothenic acid	Non-blocker	Negative	Negative	Negative	3.626
1-O-Caffeoylglycerol	Non-blocker	Negative	Negative	Negative	4.933

H-HT, human hepatotoxicity; DILI, drug induced liver injury.

**Table 3:** The evaluation of drug-likeness properties on key metabolites.

Compound	MW (g/mol)	HBA	HBD	MLog P	Lipinski’s violations	Bioavailability score	TPSA
Dihydroresveratrol	230.26	3	3	2.34	0	0.55	60.69
Lunularin	214.26	2	2	2.95	0	0.55	40.46
Dihydroglycitein	286.28	5	2	0.96	0	0.55	75.99
Palmitoylethanolamide	299.49	2	2	3.39	0	0.55	49.33
Pantothenic acid	219.23	5	4	−0.8	0	0.56	106.86
1-O-Caffeoylglycerol	254.24	6	4	−0.07	0	0.55	107.22

MW, molecular weight <500; HBA, hydrogen bond acceptor < 10; HBD, hydrogen bond donor ≤ 5; MLog P, moriguchi octanol-water partition coefficient ≤ 4.15; lipinski’sviolations ≤ 1; Bioavailability score > 0.1; TPSA, topological polar surface area <140.

### 3.14 Molecular docking

**Figure 13:**
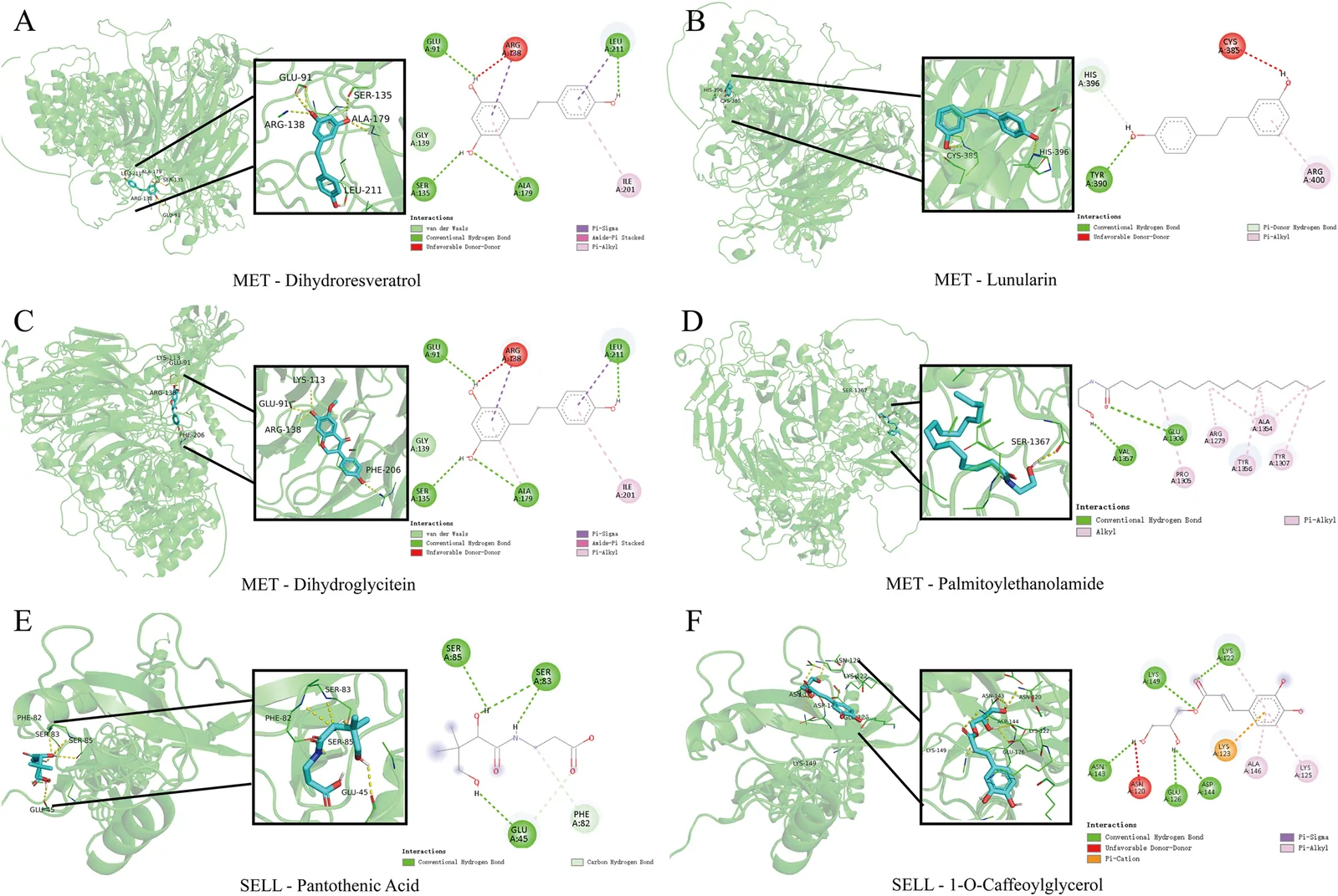
Molecular docking results. The docking analysis included the following protein-metabolite pairs: (A) MET-dihydroresveratrol, (B) MET-Lunularin, (C) MET-Dihydroglycitein, (D) MET-palmitoylethanolamide, (E) SELL-pantothenic acid and (F) SELL-1-O-caffeoylglycerol. The figure illustrates the binding interactions between MET or SELL and their respective metabolites, showing the binding conformations within the predicted active or ligand-binding pockets and the key intermolecular interactions.

Molecular docking is a method to determine the binding efficiency of a ligand in the active site of a receptor and the accompanying energy shifts. In general, if the docking energy is below 0 kcal/mol, it means the receptor and ligand can bind without external influence; lower energy levels are associated with more stable conformations and a higher likelihood of interaction. Typically, a binding energy below −5 kcal/mol is considered indicative of good binding potential. In this study, molecular docking was performed between MET and four candidate metabolites, as well as SELL and two metabolites. The results showed that all six receptor–ligand complexes exhibited certain binding capabilities (Figure 13, Table 4).

**Table 4:** Molecular docking results of metabolites and core targets.

Target	Compound	Binding energy	Binding site
MET	Dihydroresveratrol	−7.3 kcal mol^−1^	GLU-91 SER-135 ARG-138 ALA-179 LEU-211
	Lunularin	−7.1 kcal mol^−1^	CYS-385 HIS-396
	Dihydroglycitein	−6.8 kcal mol^−1^	LYS-113 GLU-91 ARG-138 PHE-206
	Palmitoylethanolamide	−5.3 kcal mol^−1^	SER-1367
SELL	Pantothenic acid	−5.1 kcal mol^−1^	PHE-82 SER-83 SER-85 GLU-45
	1-O-Caffeoylglycerol	−5.5 kcal mol^−1^	ASN-143 ASN-120 ASP-144 LYS-122 LYS-149 GLU-126

### 3.15 Molecular dynamics simulations

**Figure 14:**
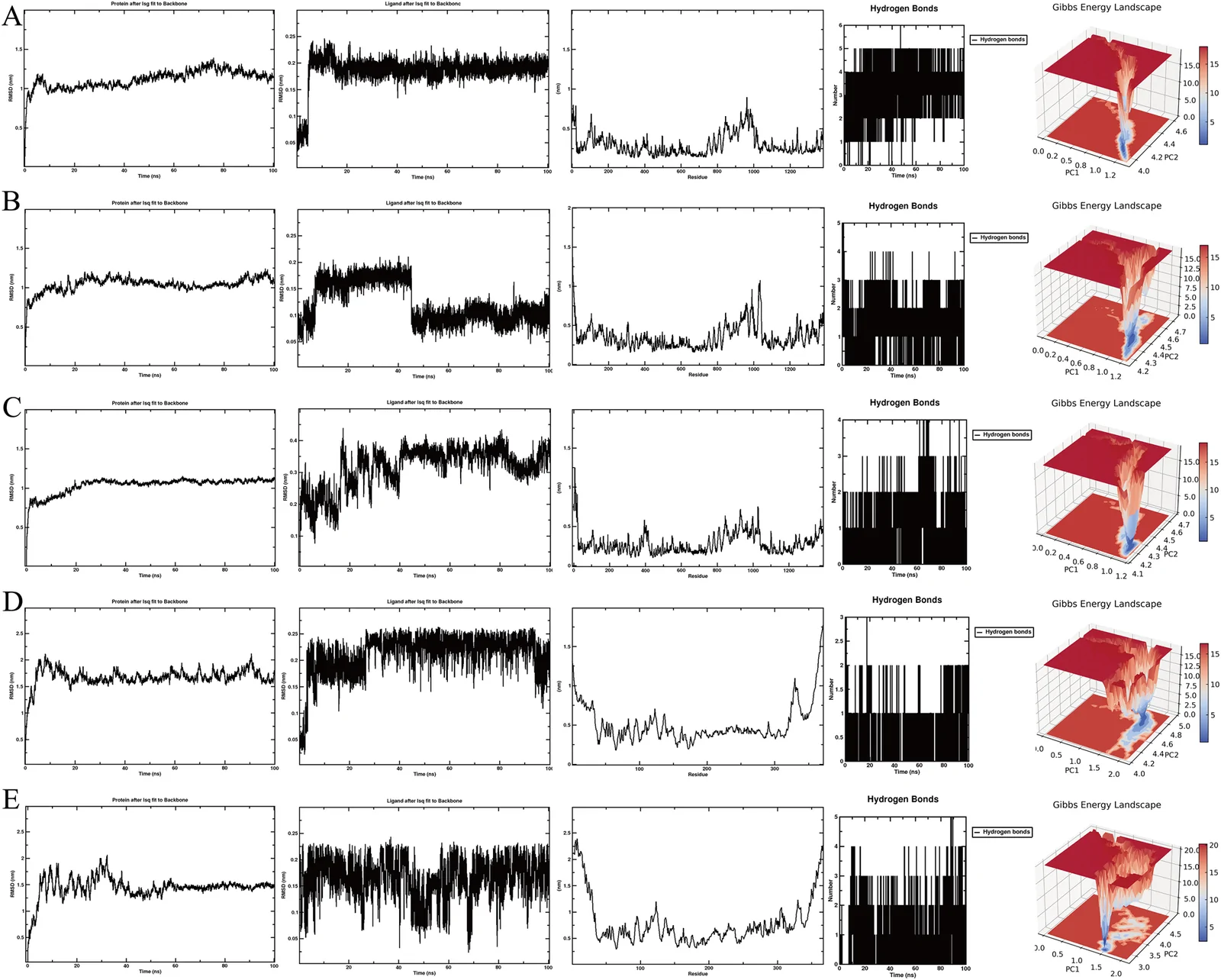
Molecular dynamics simulation results of two proteins with multiple metabolites. This figure presents the molecular dynamics simulation results of complexes formed between two proteins and several metabolites, including MET-dihydroresveratrol. (A) MET-dihydroglycitein, (B) MET-palmitoylethanolamide, (C) SELL-pantothenic acid, (D) and SELL-1-O-caffeoylglycerol and (E) each complex includes five analytical metrics: Protein backbone RMSD, ligand RMSD, protein residue RMSF, hydrogen-bond evolution, and the three-dimensional binding free-energy landscape. Together, these analyses comprehensively evaluate the overall structural stability of the proteins, the conformational dynamics of the ligands within the binding pocket, local flexibility characteristics, the stability of protein-ligand interactions, and the energy distribution defining the preferred binding conformations.

To further evaluate the binding stability and dynamic behavior of the complexes formed by the two core targets and six candidate metabolites, molecular dynamics simulations were performed. By analyzing protein backbone RMSD, ligand RMSD, residue fluctuations (RMSF), hydrogen-bonding patterns, and the Gibbs free-energy landscape, differences in conformational stability, structural flexibility, and energy states among the complexes were comprehensively assessed (Figure 14). However, based on the molecular dynamics simulation results of the MET-Lunularin complex, the protein system did not reach a stable state within 100 ns. Therefore, we conclude that Lunularin is not suitable as a drug candidate. These results provide a basis for identifying the metabolites with the strongest regulatory potential toward the core targets.

## 4 Discussion

Endometriosis is a complex disease characterized primarily by chronic inflammation, immune dysregulation, and estrogen-dependent abnormalities, with its onset and progression involving finely tuned regulatory networks across multiple systems and hierarchical levels. The role of gut microbiota in the regulation of disease onset and progression has been increasingly supported by recent evidence. As an important modulator of the host immune system, the gut microbiota can participate in and promote the initiation and progression of endometriosis by reshaping the composition and functional states of immune cells within the peritoneal cavity [29]. This study, for the first time, employed a multi-omics integration strategy to systematically investigate the potential role of gut microbiota in endometriosis from a holistic perspective of “gut microbial metabolites-host targets-immune microenvironment-disease phenotype” and established a relatively complete molecular regulatory loop.

In this study, integrated multi-cohort transcriptomic analyses identified 61 differentially expressed genes associated with gut microbiota-derived metabolites in endometriosis. By combining PPI network analysis with multiple machine learning algorithms, MET and SELL were robustly screened as core hub targets. Both genes demonstrated stable performance in terms of differential expression, network centrality, and diagnostic accuracy (AUC > 0.7). Further pQTL-based MR analysis found a significant negative association between MET and the risk of endometriosis, whereas SELL showed a positive association, suggesting MET could be protective and SELL could promote the disease. These findings suggest that MET and SELL may serve as potential therapeutic targets in endometriosis. In endometriosis research, MET and SELL are considered to play important roles in disease onset and progression. MET has been identified as a hub gene in endometriosis, showing significant expression changes in lesion tissues and suggesting a role in lesion formation via regulation of immune responses and the inflammatory microenvironment [30]. SELL (L-selectin, CD62L) mediates leukocyte tethering and rolling on vascular endothelium, initiating immune cell recruitment to inflamed tissues [31], 32]. This suggests it may influence the immune microenvironment in endometriosis, especially in inflammatory cell infiltration and immune regulation. Therefore, MET and SELL are not only associated genes in endometriosis but also potential targets for disease modulation due to their roles in immune regulation and inflammatory responses.

The present findings suggest that MET may have a potentially protective association with endometriosis, whereas SELL may be positively associated with disease susceptibility. High MET expression is enriched in pathways related to embryonic development, epithelial differentiation, and tumor-associated signaling, whereas low expression is associated with metabolic imbalance, extracellular matrix remodeling, and immune dysregulation. Immune infiltration analysis showed that MET positively correlates with M0 macrophages and activated NK cells, but negatively with pro-inflammatory M2 macrophages, suggesting that MET downregulation may promote M2 polarization, creating a pro-inflammatory and pro-fibrotic lesion microenvironment and resulting in loss of its protective function. Single-cell analysis further localized MET predominantly to epithelial cells, and CellChat analysis highlighted MIF signaling–mediated epithelial–immune cell communication, suggesting that MET maintains tissue homeostasis and immune tolerance by modulating epithelial responses to immune signals. In contrast, SELL is significantly upregulated in endometriosis, with high expression enriched in immune-inflammatory responses, complement activation, and leukocyte migration pathways. Immune infiltration analysis revealed a positive correlation between SELL and neutrophils and a negative correlation with Tregs and naïve B cells. Single-cell analysis showed widespread expression of SELL across multiple immune cell types, and CellChat analysis suggested that SELL may amplify local immune cell aggregation and inflammation through the CXCL12-CXCR4 and MIF-CD74-CXCR4/CD44 axes, thereby exacerbating lesion microenvironment imbalance and promoting disease progression.

KEGG enrichment analysis revealed that pathways associated with endometriosis mainly involve PI3K-Akt, estrogen, MAPK, Ras, Integrin, and immunoglobulin superfamily cell adhesion molecule (IgSF CAM) signaling pathways. In endometriosis, PI3K-Akt signaling promotes cell survival, migration, invasion, and angiogenesis, thereby supporting lesion proliferation and chronic inflammation [33]. Estrogen signaling regulates cell metabolism, anti-apoptotic processes, and pro-inflammatory responses [34]. The MAPK pathway is also involved in the disease pathology [35]. Integrin-mediated adhesion signaling promotes endometriosis progression via Ras/MAPK–ERK activation, regulating cell migration, invasion, and lesion establishment, whereas IgSF CAMs contribute to immune–stromal crosstalk in the inflammatory microenvironment [36]. These signaling pathways collectively regulate cell proliferation, migration, immune modulation, and inflammatory responses, representing key molecular mechanisms underlying the development and progression of endometriosis.

This study further constructed a “gut microbiota-metabolite-host target” regulatory network, revealing that multiple gut microbial metabolites can simultaneously act on MET and SELL, suggesting their central role in microbiota–host signal transmission. Specifically, the abundance of *Escherichia coli* and *Enterobacteriaceae* is increased in the gut of endometriosis patients, potentially associated with systemic inflammation and microbial dysbiosis, indicating a role in inflammatory regulation and endotoxin-driven proinflammatory mechanisms [37], [38], [39]. Changes in *Bacteroides* and *Parabacteroides* may affect host metabolism and immune regulation [40], [41], [42]. A decreased abundance of *Bifidobacterium* indicates weakened microbiota-mediated protection. This can exacerbate inflammation, impair the intestinal barrier, and disrupt hormone metabolism [43], 44]. Studies have shown that Palmitoylethanolamide (PEA) may help alleviate endometriosis-related pelvic pain. It may also reduce lesion size. In animal models, co-administration of micronized PEA and polydatin inhibited lesion development. It also improved pathological indicators. These findings suggest a potential anti-inflammatory and analgesic role for PEA in endometriosis therapy [45]. Beyond these known microbial taxa and metabolites, we also identified several novel potential microbes and metabolites that may contribute to endometriosis pathogenesis and the immune–metabolic regulatory network through similar or entirely new mechanisms, providing new directions for further functional validation and targeted intervention.

Single-cell transcriptomic analysis revealed that the MIF signaling pathway and CXCL chemokine pathways are highly active in endometriotic lesions. Specifically, MIF is highly expressed in ectopic endometrial and immune cells and may contribute to the establishment and persistence of lesions through modulation of immune cell interactions and inflammatory responses [46]. Meanwhile, the CXCL12–CXCR4 axis has been implicated in intercellular communication between endometrial cells and bone marrow-derived cells and may contribute to cellular proliferation, migration, and immune cell recruitment. Therefore, the MIF and CXCL pathways represent core nodes of immune regulation within the endometriosis microenvironment and may serve as potential therapeutic intervention targets.

Although the integrative analytical framework in this study provides new insights, several limitations remain. First, the absence of direct gut microbiome sequencing data, together with the limited coverage of gutMGene, restricts the ability to fully capture the compositional, functional, and context-specific features of gut microbial metabolism. Second, the relatively small sample size, the lack of an independent external validation cohort, and the absence of a nested cross-validation framework may increase the risk of overfitting and information leakage, thereby limiting the robustness and generalizability of the machine learning results. Third, colocalization analysis was not performed, which may limit causal inference. Additionally, multiple-testing correction was not applied due to the limited number of prioritized candidate genes. Therefore, the MR findings should be interpreted cautiously and require validation in larger genetic cohorts and experimental studies. In addition, *in vivo* endometriosis models are still required to functionally validate the metabolites and targets identified in this study, in order to clarify their causal roles and assess their therapeutic potential.

## 5 Conclusions

This study constructed an integrative multilayer network connecting core targets, microbial metabolites, and key microbial taxa in endometriosis. MET and SELL were identified as core hub genes, with MET showing a potentially protective association and SELL exhibiting a modest positive association with endometriosis risk. The gut microbiota, including *Escherichia coli, Enterobacteriaceae*, *Bacteroides*, *Parabacteroides*, and *Bifidobacterium*, together with metabolites such as palmitoylethanolamide, were associated with immune and inflammatory signaling pathways. These associations may contribute to changes in lesion formation and the local immune microenvironment. Single-cell analyses further suggested that microbial metabolites may be involved in regulating intercellular immune communication via the MIF and CXCL signaling axes. Molecular docking and molecular dynamics simulations suggested potential binding interactions and structural stability between several metabolites and core protein targets, providing structural insights for future experimental validation. Therefore, these findings suggest a potential, computationally inferred gut microbiota-metabolite-host target-immune regulatory network in endometriosis, providing a hypothesis-generating framework for future experimental validation and microbiota-targeted therapeutic studies.
